# The transcriptomic blueprint of molt in rooster using various tissues from Ginkkoridak (Korean long-tailed chicken)

**DOI:** 10.1186/s12864-021-07903-9

**Published:** 2021-08-05

**Authors:** Clémentine Charton, Dong-Jae Youm, Byung June Ko, Donghyeok Seol, Bongsang Kim, Han-Ha Chai, Dajeong Lim, Heebal Kim

**Affiliations:** 1 Department of Agricultural Biotechnology and Research Institute of Agriculture and Life Sciences, Seoul National University, Seoul, Republic of Korea; 2 eGnome, Inc, Seoul, Republic of Korea; 3 Animal Genomics & Bioinformatics Division, National Institute of Animal Science, RDA, 1500 Wanju, Republic of Korea

**Keywords:** RNA-Seq, Molt, Transcriptomics, Micro RNA, Differential gene expression, Differential transcript usage

## Abstract

**Background:**

Annual molt is a critical stage in the life cycle of birds. Although the most extensively documented aspects of molt are the renewing of plumage and the remodeling of the reproductive tract in laying hens, in chicken, molt deeply affects various tissues and physiological functions. However, with exception of the reproductive tract, the effect of molt on gene expression across the tissues known to be affected by molt has to date never been investigated. The present study aimed to decipher the transcriptomic effects of molt in Ginkkoridak, a Korean long-tailed chicken. Messenger RNA data available across 24 types of tissue samples (9 males) and a combination of mRNA and miRNA data on 10 males and 10 females blood were used.

**Results:**

The impact of molt on gene expression and gene transcript usage appeared to vary substantially across tissues types in terms of histological entities or physiological functions particularly related to nervous system. Blood was the tissue most affected by molt in terms of differentially expressed genes in both sexes, closely followed by meninges, bone marrow and heart. The effect of molt in blood appeared to differ between males and females, with a more than fivefold difference in the number of down-regulated genes between both sexes. The blueprint of molt in roosters appeared to be specific to tissues or group of tissues, with relatively few genes replicating extensively across tissues, excepted for the spliceosome genes (*U1*, *U4)* and the ribosomal proteins (*RPL21*, *RPL23)*. By integrating miRNA and mRNA data, when chickens molt, potential roles of miRNA were discovered such as regulation of neurogenesis, regulation of immunity and development of various organs. Furthermore, reliable candidate biomarkers of molt were found, which are related to cell dynamics, nervous system or immunity, processes or functions that have been shown to be extensively modulated in response to molt.

**Conclusions:**

Our results provide a comprehensive description at the scale of the whole organism deciphering the effects of molt on the transcriptome in chicken. Also, the conclusion of this study can be used as a valuable resource in transcriptome analyses of chicken in the future and provide new insights related to molt.

**Supplementary Information:**

The online version contains supplementary material available at 10.1186/s12864-021-07903-9.

## Background

Molt is a physiological event existing in multiple phyla across the animal kingdom, characterized by a periodic partial or complete shedding and replacement of the organism’s outer layer. In birds and mammals, molt is associated to a variable loss and replacement of worn appendages (feathers or hair). In birds, molt is a critical stage in the life cycle as individual fitness tightly depends on the functionality of its plumage, which, besides flight, also regulates thermoregulation and mating aptitudes. Despite its crucial influence on the organism, molt remains one of the most poorly understood and studied physiological event [1].

Renewing of plumage could be considered as the ‘tip of the iceberg’ when describing the physiological changes occurring during molt in birds. If feather loss is undoubtedly the most remarkable manifestation of molt, this process deeply affects a much wider range of tissues. Increase in metabolic rate, remodeling of the reproductive tract, transient modification of the immune system, osteoporosis, modification of proteins synthesis, decrease in body fat and increase vascularization in the dermis are all documented consequences of molt, reviewed by Kuenzel et al. 2003 [2]. The decrease or quiescence of reproductive function certainly has been the most extensively depicted physiological effect of molt because of its economic impact. Indeed, in commercial laying hens, the diminution or even cessation of eggs production occurring during molt impacts the overall profitability of the flock. More specifically, molt triggers a regression and remodeling of the ovaries and oviducts in hens. The tubular glands, the structure at the origin of the formation of the double-layered shell membrane of eggs, has been shown to degenerate and reform during molt, probably by a combination of apoptosis, autophagy and necrosis [3, 4]. Similarly, ovarian stroma has been shown to undergo a regression followed by further recrudescence [4]. Though less documented, molt appears to impact the immune system in a contrasted way/manner. On one hand, in chicken, reproductive quiescence has been shown to be associated with recrudescence and lymphotic repopulation of the thymus, and, as such, might promote immune function [5]. On the other hand, a transient decrease in the number of B cell has been shown in fasting-induced molts and might contribute in reducing immune function in response to new antigenic challenges [6]. As all the physiological changes associated with molt described in the above indicate a state of stress, it has been proposed that molt could be associated to a shift in the balance of the autonomic nervous system. More precisely, molt could correspond to a change of dominance from the parasympathetic nervous system (activated during reproduction) in the direction of the sympathetic system [2].

If the impact of molt on the organism has to date been poorly investigated in comparison to other physiological events, so have been its control and mechanisms. In many avian species, circadian and circannual rhythms are under the influence of the photoperiod. These rhythms, mediated by the neural and endocrine systems, regulate essential physiological processes such as reproduction [7, 8]. Molt occurs naturally in birds at the end of the breeding period. In domesticated chicken, though molt can be artificially induced by dietary modifications (starvation, diets deficient or unbalanced in an essential nutrient such as calcium or zinc), postnuptial molt is naturally induced by photoperiod [4]. In the jungle fowl (*Gallus gallus*), wild progenitor of the domesticated chicken, molt is initiated during broodiness [7, 9]. Accordingly, natural molt is regulated by a variety of hormones whose synthesis and secretion is influenced by seasonal effects acting on the pituitary gland (mainly through melatonin signaling and hypothalamic control) [8]. Reproductive hormones such as oestrogen and testosterone have been shown to inhibit the onset of molt whereas prolactin and the thyroid hormone thyroxine have been proved to be the most effective hormones in inducing molt [2]. Prolactin, whose concentration rise as the reproductive cycle proceeds, inhibits the hypothalamic-pituitary-gonadal axis, and particularly the secretion of gonadotropin releasing hormone (GnRH) and luteinizing hormone (LH). This rise in prolactin concentration closely matches the onset of broodiness, followed by the inactivation of the reproductive function [7]. By contrast, thyroxine is suspected to be the main responsible for the onset of feather loss and replacement by increasing metabolic activity of feather follicles, in a permissive rather than causal manner [8]. Thyroxine however does not seem to exert an influence on reproduction, so that feather loss and reproductive cycle might be under separate control [7]. While prolactin and thyroxine seem to be the hormones most intimately related to molt, a variety of other hormones interact to regulate molt: increased cortisol levels has for example been found to suppress thyroid hormones, resulting in slower and longer molt [8]. Cortisol and oestrogen have also been reported to suppress feather synthesis, in opposition to thyroxine and progesterone which promotes it [8].

Despite the potential insights that could arise from deciphering such an extensive tissue remodeling event, to date, only one study investigated molt on the gene expression level and focused on laying hens’ reproductive tract alone [4]. This present study was designed as the first big scale transcriptomics analysis of molt in chicken. Messenger RNA sequencing was performed across a total of 24 tissues issued from nine male Ginkkoridak individuals, and blood was collected on twenty individuals from both sexes. Histologically, the collected tissues encompassed the four main tissues types: nervous, connective, muscle and epithelial. Another strength of our data was the availability of both microRNA and messenger RNA sequences in blood samples. MicroRNAs are small endogenous RNAs that regulate gene expression at the posttranscriptional level, and as such, intervene in a wide range of biological processes, but a single miRNA could have up to thousands of target genes so that determining their effect on gene expression is not trivial [10–12]. Integration of miRNA data with gene expression data through inverse correlation has been shown to improve the reliability of the prediction of its effects on gene expression. In the present study, the profusion of tissues sampled and the conjoint analysis of miRNAs and mRNAs thus provided the material for an exhaustive understanding of the physiology of molt at the scale of the organism.

The breed used for the purpose of this study is named Ginkkoridak, which means “long-tailed chicken” in Korean. This domestic long-tailed chicken is native from Korea, where it is estimated to have appeared in the middle of the third century. It is listed in the Food and Agriculture Organization of the United Nations (FAO). If this breed was maintained in Korea for an extensive period, it almost disappeared in the early 1900s, shortly after the introduction of improved commercial chicken in the peninsula. On the verge of extinction, this breed was rescued thanks to the obstinacy of one farmer. As of today, about 250 Ginkkoridak are held in conservancy, and Ginkkoridak is on the process of being designated as natural monument of Korea. As its name indicate, Korean long-tailed chicken are characterized by the elongation of their tail feathers, which can grow over one meter in 1 year. On the difference of other long-tailed chicken, such as the Japanese long-tailed chicken, Onagadori, Ginkkoridak do molt every year (soft molt), after the rainy season.

The aims of this study were 1) to investigate the impact of molt on gene expression across a wide range of male Ginkkoridak tissues 2) to compare the effect of molt in male and female based on mRNA differential gene expression (DGE) and differential transcript usage (DTU) and differentially expressed miRNAs (DEM) and 3) to integrate male tissue mRNA DGE and DTU and miRNA DEM to identify potential role of miRNA and marker genes of molt.

## Results

### Overall gene expression changes during molt

**Fig. 1:**
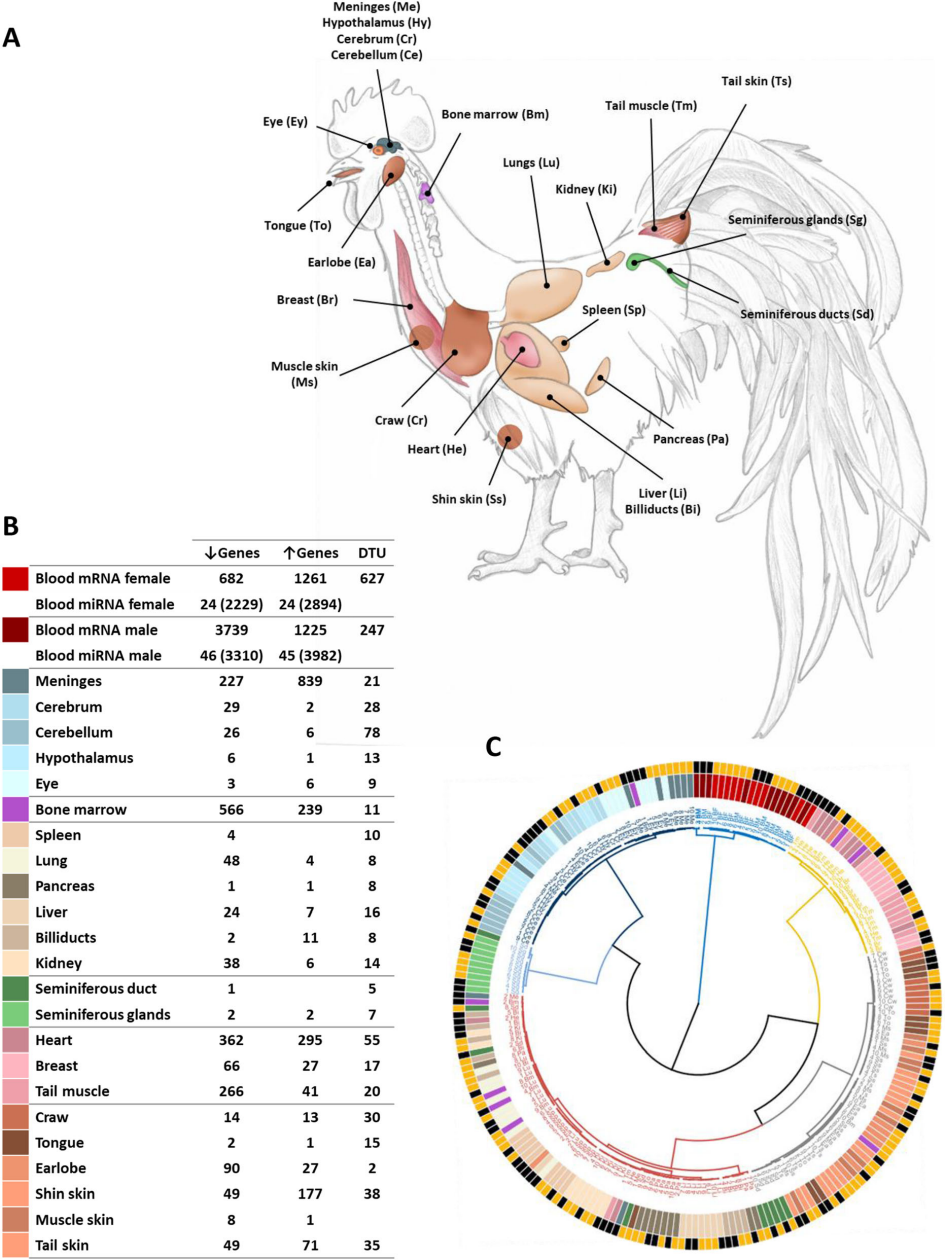
Ginkkoridak molt expression body map. **A** Map of the tissue samples. **B** Number of genes differentially expressed (over and under-expressed genes, FDR < 5%) and exhibiting differential transcript usage (DTU, OFDR < 5%). Target genes for miRNAs are indicated under brackets. **C** Clustering of the samples based on the genes differentially expressed (FDR < 5%) or exhibiting differential transcript usage (OFDR< 5%) in at least one of the tissue samples (*n* = 9538). The inner circles colors correspond to the 25 different tissues (color key in Figure 1 (**B**)). The outer circle codes for the molt status: black samples were taken before molt and orange samples were taken during molt

The present study included transcriptomic data issued from a total of twenty-five different tissues, encompassing blood, tissues from the nervous, reproductive and the digestive systems and from the skin. An overview of these tissues is displayed in Fig. 1A for concision.

The most striking observation that emerged from the analyses is that the effect of molt on transcriptome varies substantially among tissues. Most tissues did not show extensive DGE or DTU (Fig. 1B, top five genes in DGE and DTU for each tissue in Additional file 1: Table S1). Blood was the most impacted in terms of both DGE and DTU genes, followed by meninges, bone marrow, heart, tail muscle and shin skin. Most of the genes in DGE were under-expressed in response to molt with exception of the meninges, the shin and tail skins and to a lesser degree the billiducts. In blood, discrepancy was observed between sexes, though approximatively the same number of genes were up-regulated in male and female, the number of down-regulated genes in males was more than five times larger than in female. Thus molt resulted mostly in down-regulation of genes in male blood and in up-regulation in females. Although globally less genes exhibited differential transcript usage than differential expression, differential transcript usage level appeared more consistent across tissues. As for DGE, the DTU genes reached their highest level in blood, followed by cerebellum and craw. Noteworthy, in some tissues, molt seemed to trigger more changes in isoform proportion than in gene expression itself: cerebellum was the most striking example, but hypothalamus, craw, spleen, pancreas, tongue, eye and reproductive tract samples also displayed more DTU than DGE.

### Genes altered by molt across multiple tissues

**Fig. 2:**
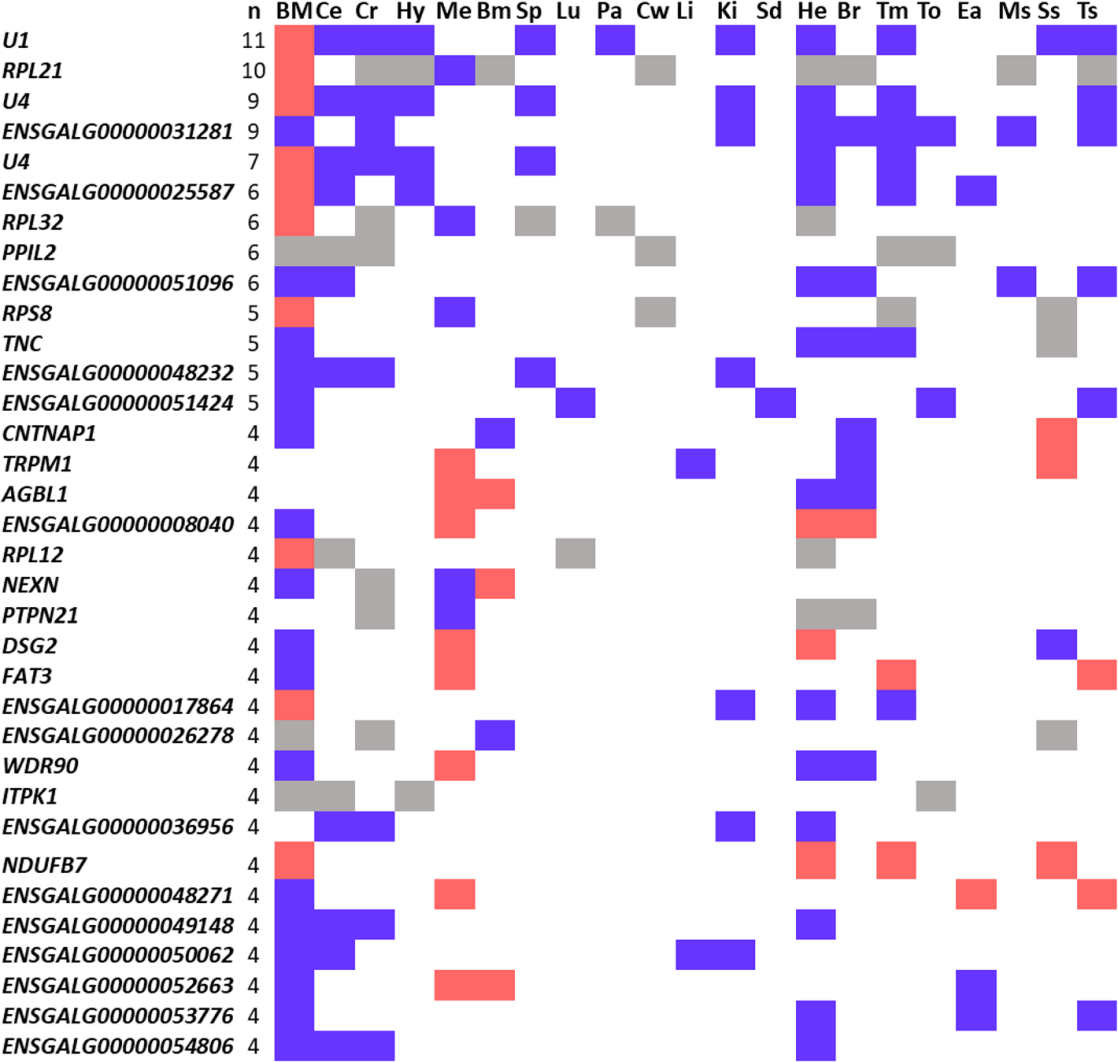
Heatmap of the genes in DGE or DTU in a least four different tissues. Genes in blue were down-regulated (FDR < 0.05), genes in red were up-regulated (FDR < 0.05) and genes in grey were in DTU (OFDR < 0.05). Tissues were referenced as follows: BM: blood in males, Ce: cerebellum, Cr: cerebrum, Hy: hypothalamus, Me: meninges, Bm: bone marrow, Sp: spleen, Lu: lungs, Pa: pancreas, Cw: craw, Li: liver, Ki: kidney, Sd: seminiferous ducts, He: heart, Br: breast muscle, Tm: tail muscle, To: tongue, Ea: earlobe, Ms.: muscle skin, Ss: shin skin and Ts: tail skin

The next step consisted in evaluating whether some genes existed that presented the same DGE or DTU profiles across various tissues during molt. However, DGE and DTU genes were for the most part found to scarcely replicate from one tissue to another, with a total of 188 genes present in at least three different tissues among the 24 collected (Additional file 2: Table S2). Among these genes, 34 were present in at least 4 tissues (Fig. 2).

The most consistently found genes were *U1* (11 tissues), *RPL21* (10 tissues), *U4* (ENSGALG00000017907) and ENSGALG00000031281 (9 tissues), *U4* (ENSGALG00000017906) (7 tissues) and ENSGALG00000025587, *RPL32*, *PPIL2* and ENSGALG00000051096 (6 tissues). The genes *U1* and *U4* code for two small nuclear (sn) RNAs whose association with the 3 other the (sn) RNAs U2, U5 and U6 and proteins forms the spliceosome, the complex mediating alternative splicing [13]. In response to molt, these genes were down in all tissue in which they were in DGE excepted for blood, in which they were up-regulated. Interestingly, the genes *RPL21* and *RPL23* that code for the ribosomal proteins L21 and L23, respectively, were found to exhibit some similar patterns in response to molt: over-expressed in blood, under-expressed in meninges and in DTU in all other tissues.

To get the most comprehensive picture from the impact of molt across chicken tissues, each of the 188 genes found to overlap in at least 3 tissues were individually assessed for their function from the literature or browsing GeneCards. When summarizing the physiological processes related to these genes, the most abundant pattern emerging was nervous system (*TNC*, 5 tissues*; CNTNAP1* and *FAT3*, 4 tissues, among others) and cells dynamics (EIF3I, *SALL1*, *GPC5*, *WNK1*, *LGMN* …). However, genes related to immunity (*JAK3*, *RSFR*, *PPIL2*, *AQP9* …), fiber and movement (*ACTN2*, *WNK1*, *TNNT1*, *AHRGAP26* …), hormones and sensing (*TEF*, *TOX2*, *NR4A2*, *GSTA3*, *MYO7A*, *BRD8*), bone formation or regeneration (*CA2*, *HIVEP3*, ENSGALG00000033591), vascular system (*ITGB8*), lipids metabolism (*PLTP*, *CEL*, *APOB*, *GYG2*, *ACSL1*) and epithelium (*EDBETA*, *CELA1* …) were also represented.

### Clustering of tissues based on the samples DGE and DTU profiles

Given the high histological and functional divergences between the tissues collected in the study, a clustering was performed in order to identify the tissues having the closest gene expression profiles. The resulting groups were then used to maximize the insights of the functional enrichment analysis. After performing a principal component analysis (PCA) on the normalized count values of the genes presenting DGE (FDR < 0.05) or DTU (OFDR < 0.05) in at least one of the tissues, samples were clustered using the HCPC function from the R package FactoMineR. A number of 6 clusters was chosen as a compromise between the inertia gain criteria. As evidenced on Fig. 1C, samples within the same tissue clustered relatively well regardless of whether the samples were taken before or during molt. The effect of molt was thus not as extreme as to disrupt the gene expression profile specific to a tissue. Clustering roughly isolated groups corresponding to the four main tissue histological types (nervous, muscle, epithelial and connective tissues), and blood and seminiferous glands were individualized into two distinct groups. Clustering first isolated blood from the other tissues. Nervous tissues and seminiferous glands samples were then individualized. Though histologically meninges is a connective tissue and eyes comprise a mixture of connective and nervous cells, they clustered closely with the nervous tissues cerebrum, cerebellum and hypothalamus. The 3 muscle tissues heart, breast and tail muscle clustered closely together. The two remaining groups, epithelial and connective tissue, were a little less homogenous in their composition. The ‘epithelial’ group gathered not only almost all skin samples (shin skin, earlobe, tail skin, muscle skin) but also samples from other organs (tongue, craw and seminiferous ducts) displaying a mixture of epithelial and muscle or connective tissues. The ‘connective tissue’ group was the most disparate in composition, gathering most of the spleen, kidney, billiducts, liver, pancreas and lungs samples, but also one earlobe, four bone marrow, two heart, one tail muscle and 2 meninges samples. The lesser cohesion found within the ‘connective’ and ‘epithelial’ tissues might originate from the fact that the tissue they include are more histologically diverse, or more might just traduce the relatively small impact of molt on most of these tissues. Lastly, the bone marrow samples stood out as being dispersed in four clusters (connective (4), nervous (1), muscle (2), and epithelial (1)).

### The impact of molt in male and female blood messenger RNA

**Fig. 3:**
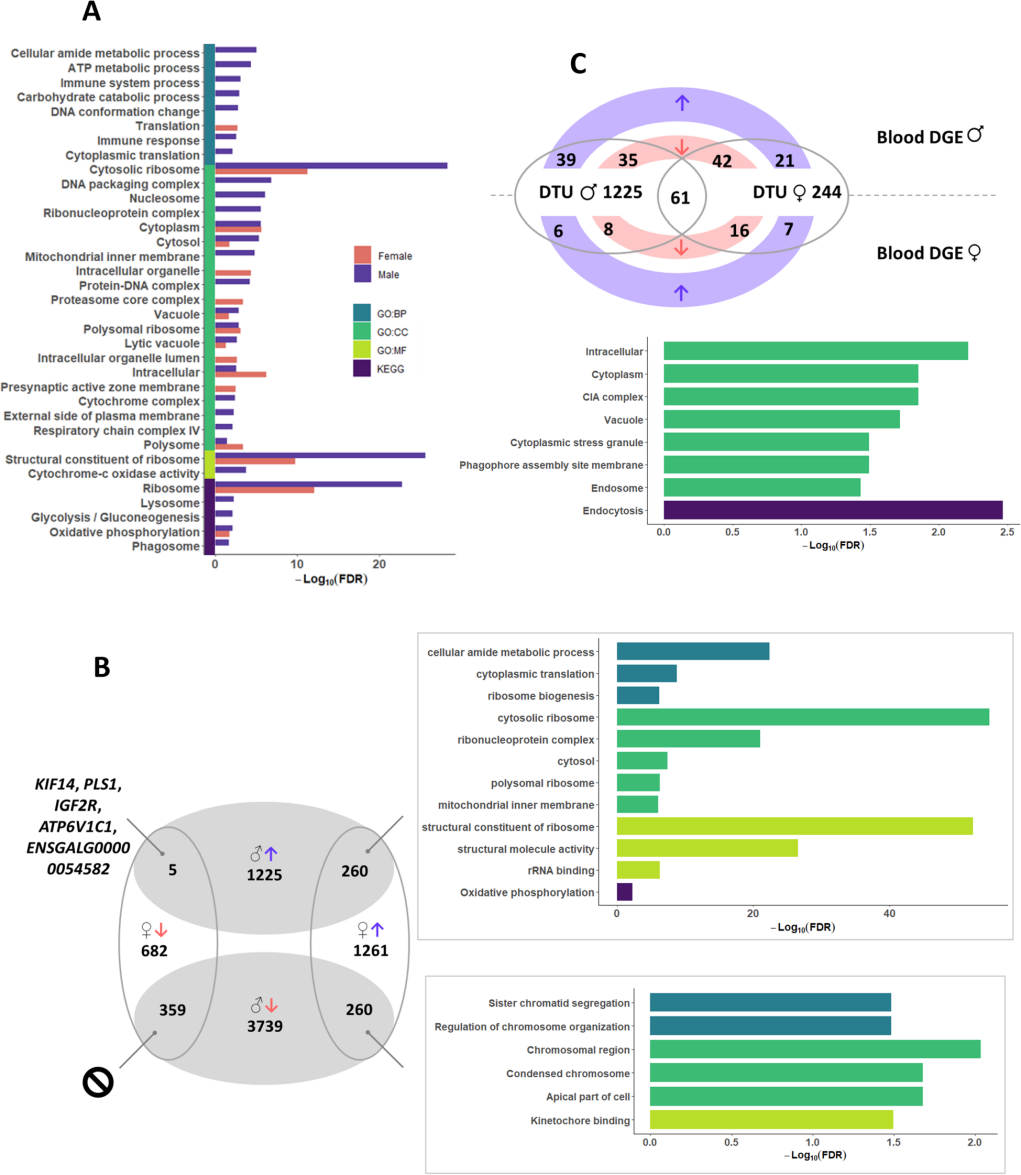
Effect of molt in male and females blood. **A** Functional enrichment analysis on male and female DGE and DTU genes. **B** Overlap between male and female over and under-expressed genes during molt and their relative functional enrichment analysis. **C** Overlap DTU between male and female and up- and down-regulated DGE genes

As briefly mentioned above, blood was the tissue most impacted by molt, the influence of molt being quantitatively different according to sex. While the number of genes over-expressed during molt and the number of DTU genes was consistent in males and females, there was a huge discrepancy in the number of down-regulated genes between the two sexes. Indeed, in males, a total of 3739 genes were shown to be down-regulated during molt, while only 682 genes were shown to be down-regulated in females (Fig. 1B, Fig. 3B). In other words, in blood, molt overall resulted in over-expression in female and in down-regulation in male.

Functional enrichment analysis was performed on genes in DGE or DTU in males and females (Fig. 3A, Additional file 3: Table S3 A-D). Only 8 GO:CC terms (‘Cytosolic ribosome’, ‘Cytoplasm’, ‘Cytosol’, ‘Vacuole’, ‘Polysomal ribosome’, ‘Lytic vacuole’, ‘Intracellular’ and ‘Polysome’) one GO:MF term (‘Structural constituent of ribosome’) and 2 KEGG pathways (‘Ribosome’ and ‘Oxydative phosphorilation’) [14] were found to be consistently enriched in both sexes. These significantly enriched terms correspond to general processes and to ribosomes. Besides these commonly enriched terms, relatively very few other GO-terms were found in female, where the term ‘Presynaptic active zone membrane’ could nevertheless indicate some alteration of the nervous system. By comparison, molt triggered in male changes in genes related to function such as immunity (‘Immune response’, ‘Phagosome’ …) or energy and metabolism (‘Mitochondrion’, ‘Respiratory chain complex IV’, ‘Glycolysis/Gluconeogenesis’ …).

The overlap DGE and DTU genes pattern between male and females is displayed in Fig. 3B. As expected from the effectives in both sexes, the biggest overlap was found for down-regulated genes, with 359 genes found common to males and females. However, no enriched terms could be found on this subset. On the contrary, 26 terms (FDR < 0.01) were found on up-regulated genes common to males and females (*n* = 260). Coherently with the earlier observation, these terms are related to translation and ribosome biosynthesis, and would mainly indicate a modification of the synthesis of the ribosomal proteins in both sexes during molt. A total of 50 genes were found to be over-expressed in females and down-regulated in males during molt. A total of 13 GO terms (FDR < 0.05) could be found enriched based on this gene list, among which ‘Kinetochore binding’, ‘Regulation of chromosome organization’, ‘Chromosomal region’ and ‘Apical part of cell’. Finally, a total of 5 genes (KIF14, PLS1, IGF2R, ATP6V1C1 and ENSGALG00000054582) were down regulated in females in up-regulated in males.

As displayed on Fig. 3C, 61 DTU genes were found to overlap in both sexes, some of them being common to DGE genes in either males or females. Gene ontology on these 61 genes evidenced 7 enriched GO:CC terms (‘Intracellular’, ‘Cytoplasm’, ‘CIA complex’, ‘Vacuole’, ‘Cytoplasmic stress granule’, ‘Phagophore assembly site membrane’ and ‘Endosome’) and one KEGG pathways (‘Endocytosis’) [14].

### The impact of molt on male tissue clusters

In order to screen for the effects of molt on the 24 tissues, functional enrichment analysis was initially considered to be done on each tissue clusters independently. This approach gave satisfying results for the epithelial cluster, for which the DGE and DTU signature was relatively consistent from one tissue to another. This approach was also used for the muscle and nervous tissues group. Indeed, although these groups presented one ‘outlier’ tissue each (meninges for the nervous cluster and heart for the muscle groups) in terms of number of DGE and DTU genes, a molt signature profile could still be identified. As a complement, a more detailed analysis was also performed on meninges and heart, respectively. For the connective group, however, DGE and DTU genes hardly replicated. This fact could result from the weakness of the DGE and DTU signal generated by molt in these tissues and by an absence of consistency in the genes impacted from one tissue to another. Lungs and kidneys, displaying more than 50 DGE and DTU genes each, were nevertheless investigated. Finally, bone marrow, that didn’t cluster with any group, was also investigated independently.

#### Epithelial tissues cluster

**Fig. 4:**
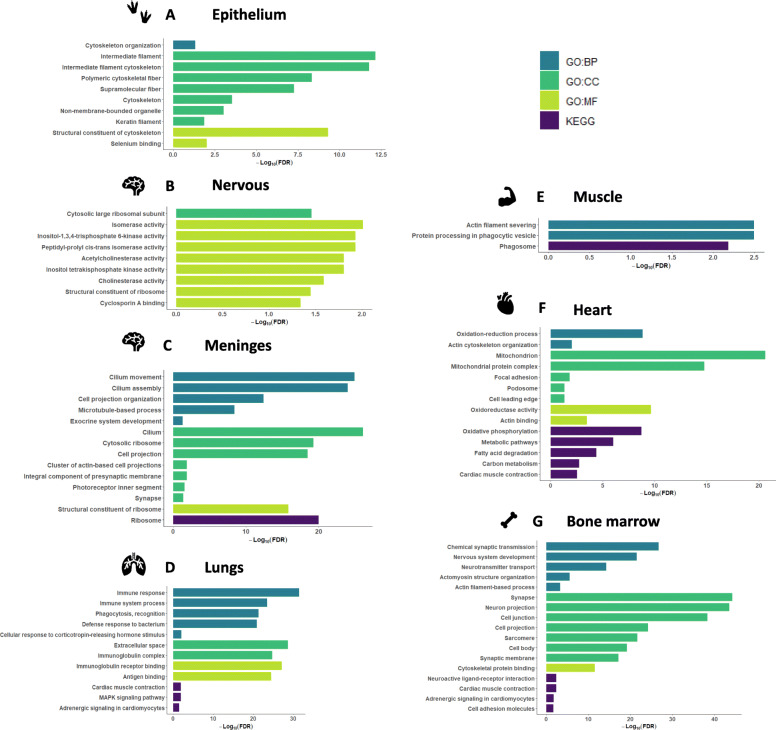
Functional enrichment analysis for each of the tissue clusters. **A** Epithelium: earlobe, craw, muscle skin, shin skin and tail skin. **B** Nervous tissues: cerebellum, cerebrum, hypothalamus and meninges. **C** Meninges. **D** Lungs. **E** Muscle tissues: heart, breast and tail muscle. **F** Heart. **G** Bone marrow

With a total of 51 DGE and DTU genes found to replicate in more than one tissue, the epithelial group presented some degree of consistency in the gene changes profile during molt. The major gene ontology results are displayed in Fig. 4A, while the complete list of enriched terms is available in Additional file 4: Table S4 A. When performing the gene set enrichment analysis on the DGE and DTU genes on tissues from the craw, the tongue, the earlobe, and the shin, muscle and tail skins, GO terms associated with a remodeling of the epithelium were found (Fig. 4A).

The epithelial tissues cluster DGE and DTU genes related to keratin, epidermis or hormones implicated in molt were further investigated (Table 1). As a result, the vast majority [15] of the genes related to keratins were found to be up-regulated in at least one of the epithelial tissues, while only 2 of these genes were down-regulated. Shin skin harbored most of these expression changes, while no genes related to keratins were found impacted in the skin muscle tissue. Tail skin and earlobe harbored the expression changes in 14 and 11 keratin-related genes, respectively. A total of 3 genes were found to be commonly up-regulated in earlobe, shin and tail skins. Two of them mapped to scale keratin-like gene while the last one, *EDBETA* (epidermal protein differentiation beta) is part of the epidermal differentiation complex (EDC) gene cluster. Noteworthy, two other genes from this cluster, *EDCH3* and epidermal differentiation protein starting with MTF motif 3-like were also among the highest overexpressed genes in tail skin tissue, epidermal differentiation protein starting with MTF motif 3-like reaching a fold change as high as 24.84. Surprisingly, molt resulted in the down regulation of beta-keratin in shin skin (fold change − 2.42). Other down regulated keratin-related in epithelium genes include one of the feather keratin 1-like related genes (ENSGALG00000049852) in the earlobe. Most of the keratins up-regulated by molt are characterized as beta keratins, and they further map to 3 out of the 4 known beta keratin groups: claw, feather and scale beta-keratins. Feather keratin are particularly well represented, with a total of 23 genes differentially expressed during molt. In proportion, alpha keratins seemed relatively less altered by molt, with 7 genes impacted. Epithelial tissue-related signature of molt also evidenced over expression of genes related to skin and feather pigmentation (*MLPH*), skin structure (collagen type XXIII alpha 1 chain). Interestingly, expression of genes related to hormones (*CRH*, *PTHLH*) and to circadian clock (*PER2*, *CRY2*) was also increased in shin skin during molt.

**Table 1:** Genes which expression pattern (DGE or DTU) has been altered by molt in at least one tissue among the epithelial cluster and whose function was putatively related to appendages

Gene ID	Gene description	n	Cw	Ea	Ss	Ts
ENSGALG00000033381	768,880 KRT75L1 keratin, type II cytoskeletal 75-like 2	2		5.23	6.93	
ENSGALG00000024136	beta-keratin	1			−2.42	
ENSGALG00000026964	claw keratin-like	1			11.72	
ENSGALG00000027475	claw keratin-like	1			13.27	
ENSGALG00000047111	claw keratin-like	1			11.62	
ENSGALG00000048696	claw keratin-like	1			10.46	
ENSGALG00000052035	claw keratin-like	1			11.18	
EDBETA	epidermal differentiation protein beta	3		6.86	8.68	7.53
EDCH3	epidermal differentiation protein containing cysteine histidine motifs 3	1				21.20
ENSGALG00000047689	epidermal differentiation protein starting with MTF motif 3-like	2			12.86	24.84
ENSGALG00000026754	feather keratin 1-like	2	1.80		5.62	
ENSGALG00000048841	feather keratin 1-like	2			11.93	6.63
ENSGALG00000051914	feather keratin 1-like	2		22.16	13.84	
ENSGALG00000026987	feather keratin 1-like	1			27.12	
ENSGALG00000027167	feather keratin 1-like	1			10.21	
ENSGALG00000028843	feather keratin 1-like	1			15.82	
ENSGALG00000038424	feather keratin 1-like	1			11.03	
ENSGALG00000047672	feather keratin 1-like	1			15.56	
ENSGALG00000049852	feather keratin 1-like	1		−6.52		
ENSGALG00000050510	feather keratin 1-like	1			23.71	
ENSGALG00000053111	feather keratin 1-like	1			13.49	
ENSGALG00000054492	feather keratin 1-like	1			13.53	
ENSGALG00000047809	feather keratin 2-like	1		23.98		
ENSGALG00000027640	feather keratin 3-like	1			27.76	
ENSGALG00000046999	feather keratin Cos1–1/Cos1–3/Cos2–1-like	1			23.57	
ENSGALG00000047404	feather keratin Cos1–1/Cos1–3/Cos2–1-like	1			25.64	
ENSGALG00000047476	feather keratin Cos1–1/Cos1–3/Cos2–1-like	1			23.82	
FK27	feather keratin Cos1–1/Cos1–3/Cos2–1-like	1			23.13	
ENSGALG00000049685	feather keratin Cos1–1/Cos1–3/Cos2–1-like	1			24.93	
ENSGALG00000050377	feather keratin Cos1–1/Cos1–3/Cos2–1-like	1			23.94	
ENSGALG00000052341	feather keratin Cos1–1/Cos1–3/Cos2–1-like	1			22.90	
FK27	feather keratin Cos1–1/Cos1–3/Cos2–1-like	1			24.61	
F-KER	feather keratin I	1			13.92	
FBN3	fibrillin 3	2		5.35	5.90	
ENSGALG00000054020	*Gallus gallus* feather keratin 3-like (LOC100859191), mRNA.	1			15.09	
ENSGALG00000024138	keratin	1			11.85	
ENSGALG00000028374	keratin	1			12.12	
ENSGALG00000039987	keratin A	1			13.66	
KRTAP10–4	keratin associated protein 10–4	1			11.51	
ENSGALG00000034054	keratin, type I cytoskeletal 42-like	1		5.81		
ENSGALG00000041858	keratin, type I cytoskeletal 42-like	1			2.02	
ENSGALG00000044875	keratin, type II cytoskeletal 75-like 4	2			10.91	8.13
ENSGALG00000053966	keratin-associated protein 10–4-like	2		23.75	11.69	
EDMPN2	keratin-associated protein 5–1-like	1			13.16	
ENSGALG00000054721	keratin-associated protein 9–1-like	1			27.22	
ENSGALG00000029144	scale keratin-like	3		8.25	7.71	6.47
ENSGALG00000033213	scale keratin-like	3		7.56	11.24	8.58
ENSGALG00000034308	scale keratin-like	2			13.75	10.41
ENSGALG00000041483	scale keratin-like	2			15.21	9.90
ENSGALG00000046792	scale keratin-like	2			9.46	22.93
ENSGALG00000052180	scale keratin-like	2			10.86	23.60
ENSGALG00000040622	scale keratin-like	1			10.92	
ENSGALG00000047022	scale keratin-like	1			9.32	
ENSGALG00000049037	scale keratin-like	1			13.41	
ENSGALG00000053010	scale keratin-like	1			11.98	
ENSGALG00000054186	scale keratin-like	1			9.15	
ENSGALG00000043660	trichohyalin-like	1			10.57	
ENSGALG00000047783	uncharacterized LOC107055273	2		22.99	25.70	
MLPH	melanophilin	1			6.40	
ENSGALG00000006708	collagen type XXIII alpha 1 chain	1			2.42	
CRH	corticotropin releasing hormone	1			5.70	
PTHLH	parathyroid hormone like hormone	1			4.16	
PER2	period circadian clock 2	1		1.61		
CRY2	cryptochrome circadian regulator 2	2		1.16		

The values are expressed as the log2 fold change of FDR. (n: number of tissues in which the gene was altered, *Cw* craw, *To* tongue, *Ea* earlobe, *Ss* shin skin, *Ms.* muscle skin and Ts: tail skin)

#### Nervous tissues cluster

Molt impacted the expression of a total of 27 genes in more than one tissue within the cluster, among which 4 were found to be affected in three tissues. Most of these altered genes were found common to the cerebrum and the cerebellum, DTU genes being extensively represented in the cerebellum. These 4 genes are the aforementioned genes related to translation regulation processes that presented the widest overlap over tissues: *U4* (ENSGALG00000017906 and ENSGALG00000017907), *RPL21* and *U1*. The U4 and U1 spliceosomal RNAs presented a similar down-regulation pattern in cerebrum, cerebellum and hypothalamus. The RPL21 gene, coding for the ribosomal protein L21, was down-regulated in the meninges and presented DTU in the cerebellum and hypothalamus. Gene ontology performed on these 27 genes evidenced 14 GO:MF and one GO:CC terms (Fig. 4B, Additional file 4: Table S4 B). If two terms related to ribosome were significantly enriched, the most striking pattern was 5 terms related to inositol metabolism, which plays a role in the development of the neural tube. Cholinesterase and acetylcholinesterase activities were also significantly enriched, implying an impact of molt in the regulation of synaptic transmission or a possible shift of the autonomic nervous system [16, 17]. More surprisingly, the GO:MF term ‘Cyclosporin A binding’, related to the immunosuppressant cyclosporin was also found enriched. Finally, the enrichment of isomerase activity related terms indicated that molt might regulate the conversion of some molecules’ isomer to other isomers in nervous tissues. As aforementioned, with 1087 DGE and DTU genes, meninges presented an outlier profile in response to molt. The gene set enrichment analysis performed on this tissue alone evidenced 32 enriched GO terms (FDR < 0.01) and 1 KEGG pathway [14] (Fig. 4C, Additional file 4: Table S4 C). Some of these terms were related to translation and ribosome, as was the case for most tissues. Others terms, however, seem coherent with a remodeling of the nervous system, in a straightforward manner like ‘Integral component of presynaptic membrane’, ‘Synapse’ (GO:CC, FDR < 0.05) or indirectly like ‘Cell projection organization’, ‘Cilium assembly’ (GO:BP) that refer to processes that can be associated to axonogenesis [18]. Lastly, the GO:BP and GO:CC terms ‘Exocrine system development’ and ‘Photoreceptor inner segment’ (FDR < 0.05) might be related to the hormonal control of molt, which is known to be triggered by photoperiod [4].

#### Muscle tissue cluster

Three tissues (breast, tail muscle and heart) were included in the cluster. Overall, molt affected muscle tissues more and more homogeneously than other tissues: 70 DGE and DTU genes could be observed for at least two of the three tissues. However, only 4 enriched GO:BP terms and one KEGG pathways [14] (FDR < 0.01) could be identified on the basis of these replicated genes. The majority of these enriched terms (3 GO:BP and one KEGG pathways, Fig. 4E, Additional file 4: Table S4 E) [14] was related to phagosome, while one GO:BP term ‘Actin filament severing’ suggests a possible impact of molt on muscle cells motility [19, 20].

If muscles related DGE and DTU genes replicated quite well compared to other tissues, heart nevertheless stood out based on the number of DGE and DTU genes identified (*n* = 712). This higher impact of molt on the cardiac muscle might derivate from its singularities compared to the skeletal muscles sampled from the breast and tail. When independently investigate, a total of 34 GO terms and 5 KEGG pathways [14] were found as significantly enriched (FDR < 0.01) in heart (Fig. 4F, Additional file 4: Table S4 F). In coherence with the tissue’s origin, most selected terms were related either to energy processes or to actin and myosin, the filaments whose movement relative to one another is at the core of muscle contraction [21]. The explicit KEGG pathways ‘Cardiac muscle contraction’ [14] was also found significantly enriched. Finally, the co-presence of terms such as ‘Cell leading edge’, ‘Podosome’, ‘Focal adhesion’ (FDR < 0.05) and ‘Actin skeleton organization’ also suggest an impact of molt on the so-called ‘cell crawling’ process, a basic form of cell locomotion notably occurring during development and wound healing [21, 22].

#### Connective tissue cluster

As mentioned earlier, with only 9 genes found in at least 2 tissues from the cluster, DGE and DTU genes hardly if ever replicated in the ‘connective’ group, probably due to the diversity of tissues this cluster encompasses. Kidney and lung, having more than 50 DGE and DTU genes, were individually investigated. Gene functional enrichment analysis performed on kidney evidenced 7 GO:MF term (FDR < 0.05) related to two genes, *SIVA1*, implicated in cell cycle progression and apoptosis, and *PLTP* coding for a protein involved in the transfer of phospholipids. By contrast, the lung gene ontology evidenced numerous terms related to immunity, among which the GO:MF term ‘Immunoglobulin receptor binding’, the GO:BP terms ‘Immune response’, ‘Phagocytosis, recognition’, ‘Defense response to bacterium’, the GO:CC term ‘Immunoglobulin complex’ and the KEGG pathways ‘MAPK signaling pathways’ [14] (FDR < 0.05) (Fig. 4D, Additional file 4: Table S4 D). Importantly, the intersection sizes between the input gene list and the terms list were large, leading to adjusted *p*-values as small as 3.65 × 10–32 and 7.49 × 10–28 for the GO:BP term ‘Immune response’ and the GO:MF term ‘Immunoglobulin receptor binding’, respectively. Though further inference of the effect of molt on lung immunity status would be overstating, it should still be noted that the vast majority of the DGE genes in lung were down-regulated in response to molt (48 down regulated for 4 up-regulated genes). Also of interest here, the KEGG pathways related to the cardiac muscle ‘Cardiac muscle contraction’ and ‘Adrenergic signaling in cardomyocytes’ [14] were also significantly enriched (FDR < 0.05). Finally, the GO:BP term “Cellular response to corticotropin-releasing hormone stimulus’ was also found significantly enriched. Interestingly, a gene linked to this stress response mediating hormone was also found over-expressed in shin skin.

#### Bone marrow

Finally, bone marrow, the tissue that didn’t clearly cluster with any of the other tissues, was investigated independently (Fig. 4G, Additional file 4: Table S4 G). This tissue appeared to have a gene functional enrichment profile at the interface between the nervous, muscle and lung tissues. Here, the impact of molt on the enrichment of nervous system related terms is even more obvious than it was on the nervous tissues and meninges. GO:CC terms like ‘Synapse’ and ‘Neuron projection’ reached adjusted *p*-values as low as 5.60 × 10–45 and 2.92 × 10–44, respectively and GO:BP terms like ‘Chemical synaptic transmission and ‘Nervous system development’ were associated with adjusted p-values of 1.93 x 10-27 and 2.93 x 10-22, respectively. As was the case for muscle tissues, terms related to actin (‘Actin filament-based process’) and actomyosin (‘Actomyosin structure organization’) were also found enriched. Finally, the two KEGG pathways that were found enriched in the lungs, ‘Cardiac muscle contraction’ and ‘Adrenergic signaling in cardiomyocytes’ [14] were also found enriched in bone marrow.

### Identification of differentially expressed micro RNAs associated with molt in blood

**Fig. 5:**
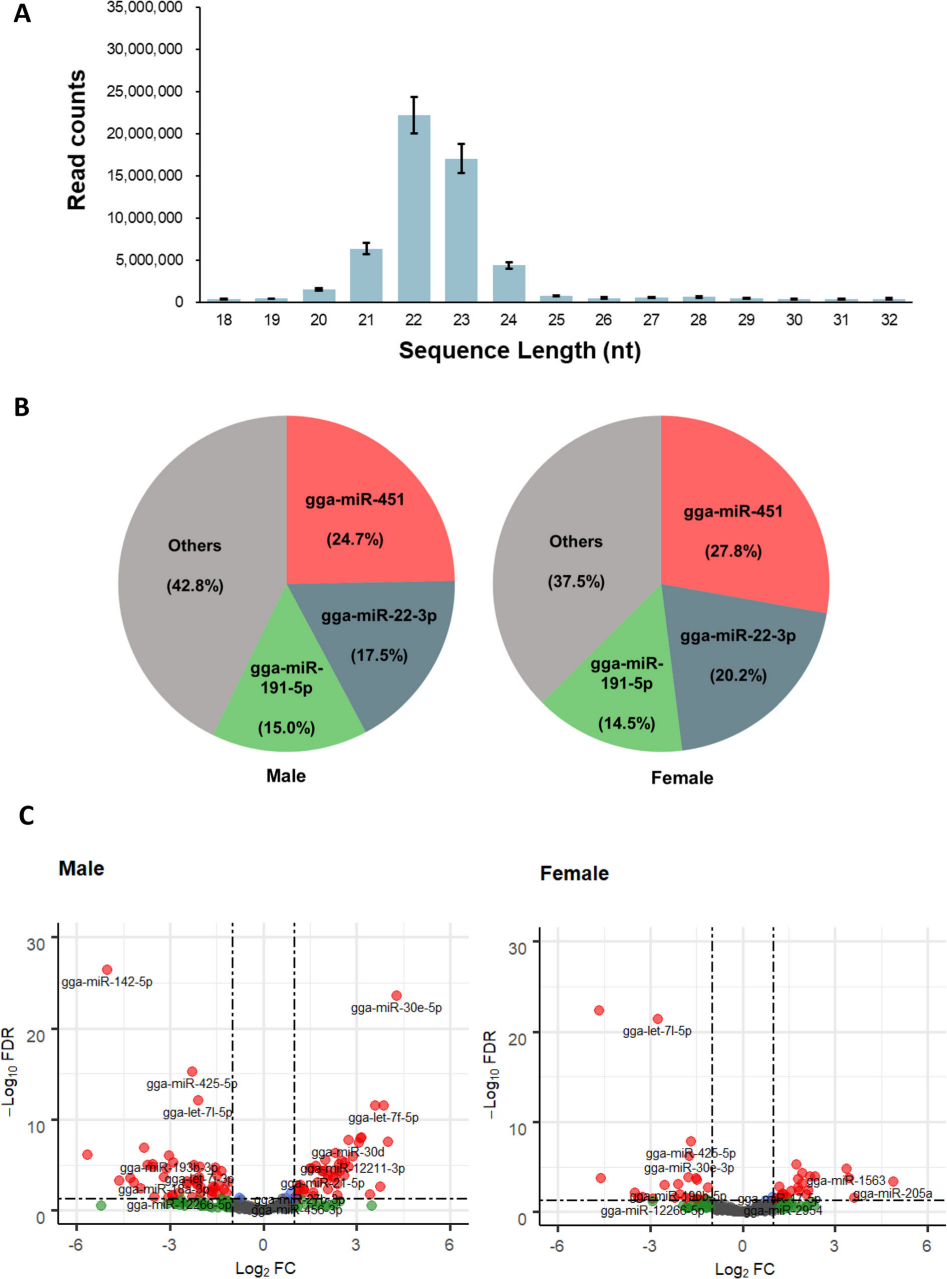
Identification and profile of miRNA in blood. **A** The average length distribution of clean reads in all samples. The error bar represents standard error (SE). **B** Pie chart of the 3 most abundantly expressed miRNAs and their respective percentages. **C** Volcano plot of DEM analysis in blood. Black dots indicate statistically non-significant DEMs, green dots indicate DEMs that meet the criteria of log2|FC| > 1 and blue dots indicate DEMs that meet the criteria of Q-value< 0.05. Red dots indicate DEMs that meet the criteria of both log2|FC| > 1 and Q-value< 0.05, which represents statistically significant DEMs

The micro RNA libraries were built from the blood samples collected in the 10 male and 10 female Ginkkoridak sampled before and during molt. In total, 1,368,526,827 reads were generated from NGS of the 20 libraries. A total of 1,110,146,504 reads (82%) with length ranging from 18 to 32 nucleotides and associated with a phred score > 30 remained after adaptor trimming, discarding of rRNA and size selection. Overall, 85.7% (950,906,789 reads) of the remaining reads could be mapped to Galgal6 (assembly GRCg6a) (Additional file 5: Table S5 A). Pre-processing was considered effective as the length distribution of the retained reads indicated that most reads were around 22 nucleotides long (Fig. 5A, Additional file 5: Table S5 B).

#### miRNA transcriptome profile

A total of 287 known chicken miRNAs were identified based on all samples (Additional file 6: Table S6 A). Representing 24.7 and 27.8% of all known miRNA’s normalized counts in male and female, gga-miR-451 was the most abundant miRNA. Taken together, the three miRNAs with the highest expression (gga-miR-451, gga-miR-22-3p and gga-miR-191-5p) represented up to 57.2 and 62.5% of the total identified miRNA reads in both male and female, respectively (Fig. 5B).

#### Differentially expressed miRNA and target gene prediction

The DEM analysis conducted in male and female Ginkkoridak aimed to identify key miRNAs associated with molt. The top 10 DEMs in male and female chicken are displayed in Table 2. A total of 91 DEMs were identified in males, out of which 45 were up-regulated and 46 were down-regulated during molt. By contrast, only 48 DEMs were identified in females, out of which 24 were up-regulated and 24 were down-regulated during molt (Fig. 5C, Additional file 6: Table S6 B). With a log2 fold change values of − 5 and − 4.6, and *q*-values of 4.30E-27 and 4.47E-23 in males and females respectively, gga-miR-142-5p was the most differentially expressed miRNA in both sexes.

**Table 2:** Top 5 significant over and under expressed miRNAs in male and female on before molt and during molt

miRNA	log2FoldChange	Q-value	Expression
**Male**			
gga-miR-142-5p	−5.021141184	4.30E-27	Under
gga-miR-30e-5p	4.273907996	3.00E-24	Over
gga-miR-425-5p	−2.279331027	6.49E-16	Under
gga-let-7 l-5p	−2.078074221	8.88E-13	Under
gga-miR-20a-5p	3.583762266	3.00E-12	Over
gga-let-7f-5p	3.869245	3.00E-12	Over
gga-miR-16-5p	3.144420726	9.31E-09	Over
gga-miR-101–3p	3.116289471	1.13E-08	Over
gga-let-7a-3p	−3.837624884	1.47E-07	Under
gga-miR-16-2-3p	−5.643369585	7.62E-07	Under
**Female**			
gga-miR-142-5p	−4.654671595	4.47E-23	Under
gga-let-7 l-5p	−2.752899118	4.25E-22	Under
gga-miR-425-5p	−1.685047714	1.49E-08	Under
gga-miR-30e-3p	−1.723825508	6.29E-07	Under
gga-miR-106-5p	1.757474057	6.01E-06	Over
gga-miR-1563	3.371644505	1.70E-05	Over
gga-let-7c-5p	1.944583596	4.91E-05	Over
gga-miR-142-3p	2.191681282	0.000108962	Over
gga-miR-30a-5p	2.356267028	0.000108962	Over
gga-miR-30c-1-3p	−1.748014943	0.000144434	Under

The mRNAs potentially targeted by the DEMs were then identified through target prediction analysis. In males, a total of 7866 potential mRNAs were predicted as target genes (4240 target genes for the 45 up-regulated DEMs and 3626 target genes corresponding to the 46 down-regulated DEMs). With a total of 5457 mRNA predicted, slightly less target genes could be predicted in females (3085 and 2372 target genes associated to the 24 up and the 24 down-regulated DEMs, respectively (Additional file 7: Table S7).

#### Integration of DEM and DGE data in blood

Targets genes issued from differentially expressed miRNAs were integrated with differential gene expression data in order to get a comprehensive view of miRNA and mRNA during molt. Since miRNAs act by repressing post-transcription of mRNA, only inversely expressed DEMs and DGE genes were considered in this study (Additional file 8: Table S8 A). Significance of overlaps was evaluated by hypergeometric test with confidence interval of 95%.

**Fig. 6:**
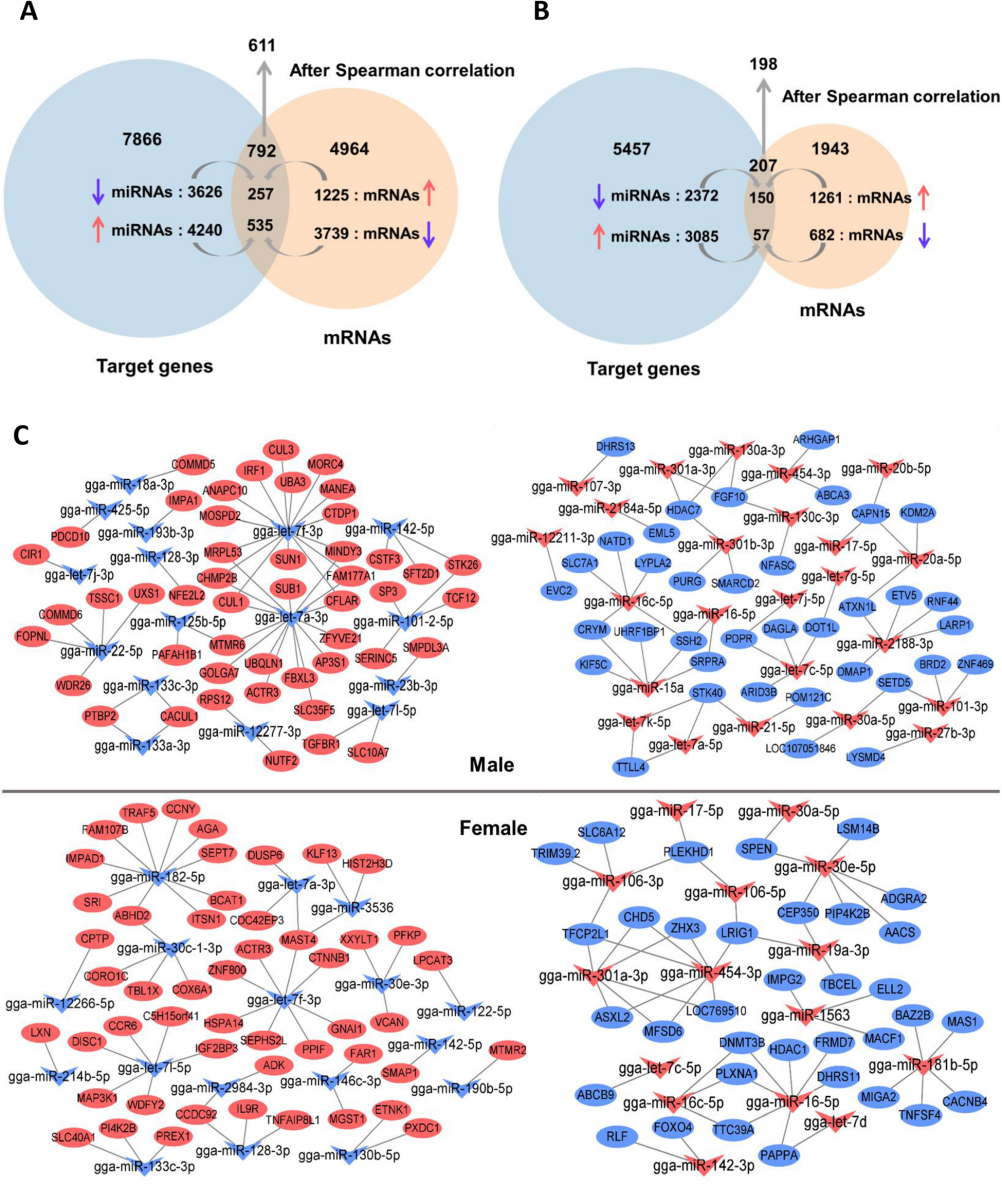
Data integration of miRNA and mRNA in blood. **A** Venn diagram of the potential miRNA target genes, DGE genes and overlap genes in male chicken. **B** Venn diagram of the potential miRNA target genes, DGE genes and overlap genes in female chicken. **C** Top 50 significant miRNA-mRNA interaction pairs in both male and female after Spearman’s rank correlation analysis. The shapes Ellipse and V represents mRNAs and miRNAs, respectively. Up-regulated genes are represented in red and down-regulated genes are represented in blue

As displayed in Fig. 6A, in male chicken, a total of 535 genes were found to overlap between the 4240 targets genes issued from the up-regulated miRNAs and the 3739 down-regulated mRNAs (*P*-value = 1.810e-13), corresponding to 1826 miRNA-mRNA pairs. Reversely, 257 genes (439 miRNA-mRNA pairs) were found to intersect between the 3626 target genes issued from the down regulated miRNAs and the 1225 up-regulated mRNAs (*P*-value = 4.163e-07). In total, 792 overlap genes and 2265 miRNA-mRNA pairs were found in male chicken blood. In females, 57 genes were found at the intersection between the 3085 target genes of the up-regulated DEMS and the 682 down-regulated miRNA (*P*-value = 2.074e-05), corresponding to 130 miRNA-mRNA pairs, while 150 genes (214 miRNA-mRNA pairs) were found to overlap between the 2372 down-regulated miRNAs target genes and the 1261 up-regulated mRNAs (*P*-value = 0.034) (Fig. 6B). In total, 207 overlap genes, corresponding to 344 miRNA-mRNA pairs were identified in females’ blood.

#### Identification of significant miRNA-mRNA pairs

Significance of miRNA-mRNA pairs was assessed based on a Spearman rank correlation analysis (Spearman rho, *q*-value < 0.05) conducted on the normalized counts of miRNAs and mRNAs. The genes from the significant miRNA-mRNA pairs, referred to as ‘overlap genes’ were retained for further analysis (Additional file 8: Table S8 B). A total of 1532 significant miRNA-mRNA pairs, corresponding to 611 overlap genes, were found in males (Fig. 6A), while 313 significant miRNA-mRNA pairs (198 overlap genes) were discovered in females (Fig. 6B). The top 50 significant miRNA-mRNA pairs for up and down-regulated DGE genes in both male and female is displayed in Fig. 6C.

#### Functional enrichment analysis of miRNA-mRNA pairs

**Fig. 7:**
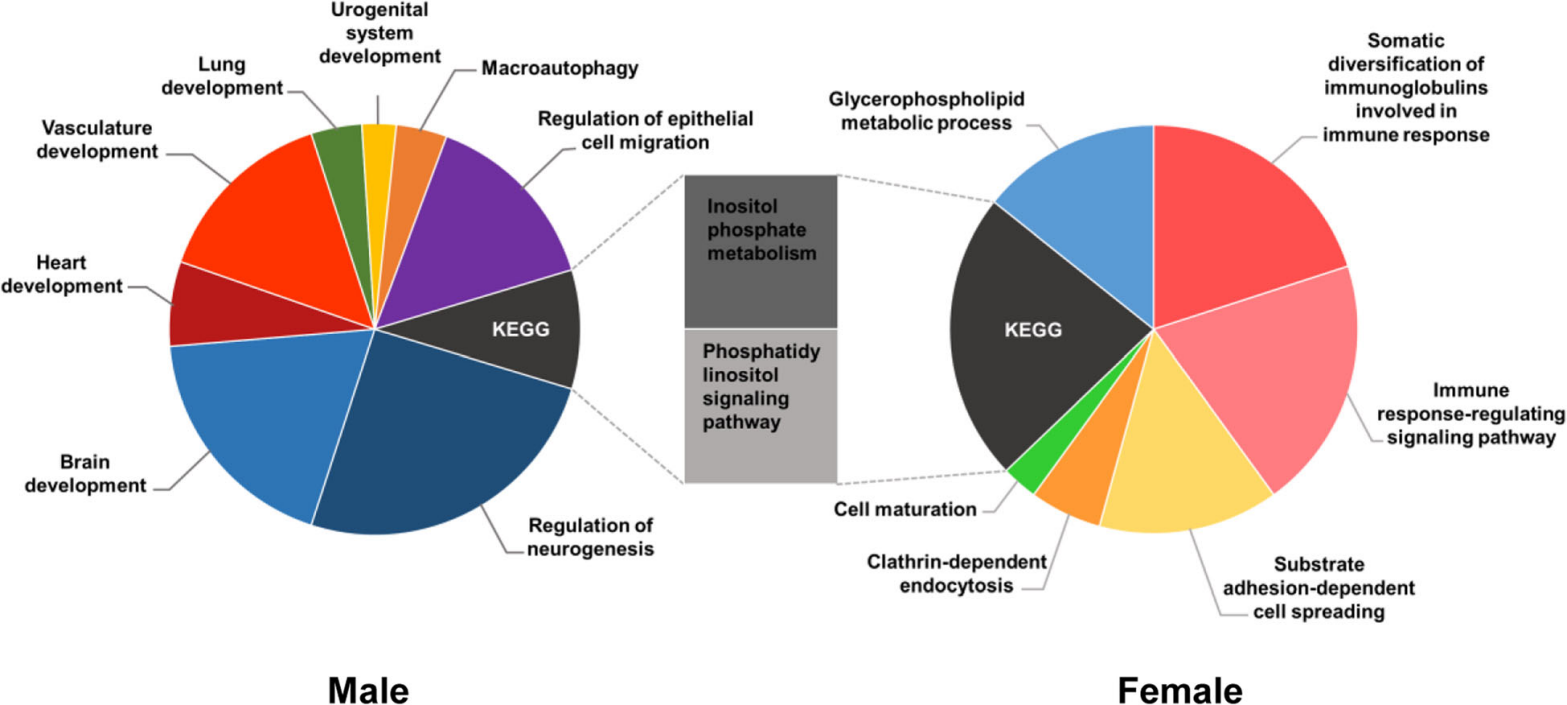
Categorization of major GO terms and KEGG pathways. Clustering analysis was conducted through ClueGO [23] in Cytoscape. An area of each section represents the relative quantity of the GO terms or KEGG pathways [14] that pertain to its major category

Functional enrichment analysis was performed in order to uncover the potential role of the DEMs in mediating the effect of molt effect and to discern the function of the overlap genes issued from the significant miRNA-mRNA pairs. Around twice as much GO terms were found in males (177) compared to females (87), while the number of KEGG pathways [14] were almost similar (7 and 8 for males and females, respectively) between sexes (Additional file 9: Table S9). This discrepancy in the number of GO terms showed on the profile of the GO terms. Indeed, GO terms in female were mostly related to immune responses (‘Somatic diversification of immunoglobulins involved in immune response’, ‘Immune response-regulating signaling pathway’) or cell dynamics (‘Cell maturation’, Substrate adhesion-dependent cell spreading’), while males GO terms were associated mostly associated to functions related to the nervous systems remodeling (‘Regulation of neurogenesis’, ‘Cerebral cortex tangential migration’, ‘Olfactory lobe development’) and to the development of various tissues and cells dynamics (‘Brain development’, ‘Heart development’, ‘Vasculature development’, ‘Lung development’, ‘Urogenital system development’, ‘Regulation of epithelial cell migration’) (Fig. 7). Interestingly, KEGG pathways were found to partly overlap between males and females with two items related to inositol metabolism, ‘Phosphatidylinositol signaling system’ and ‘Inositol phosphate metabolism’, [14] being significantly enriched in both sexes, indicating that, regardless of sex, miRNAs modulate these pathways during molt. Inositol metabolism related terms were also found to be enriched under the effect of molt in the nervous tissues cluster. As a complement, a KEGG pathway related to autophagy was also found enriched in males, while ‘Progesterone-mediated oocyte maturation’ was found enriched in females. [14] The latter seems congruent, knowing that molt, in hens, induces a whole remodeling of the reproductive tract [4].

### Data integration: candidate biomarkers of molt

**Fig. 8:**
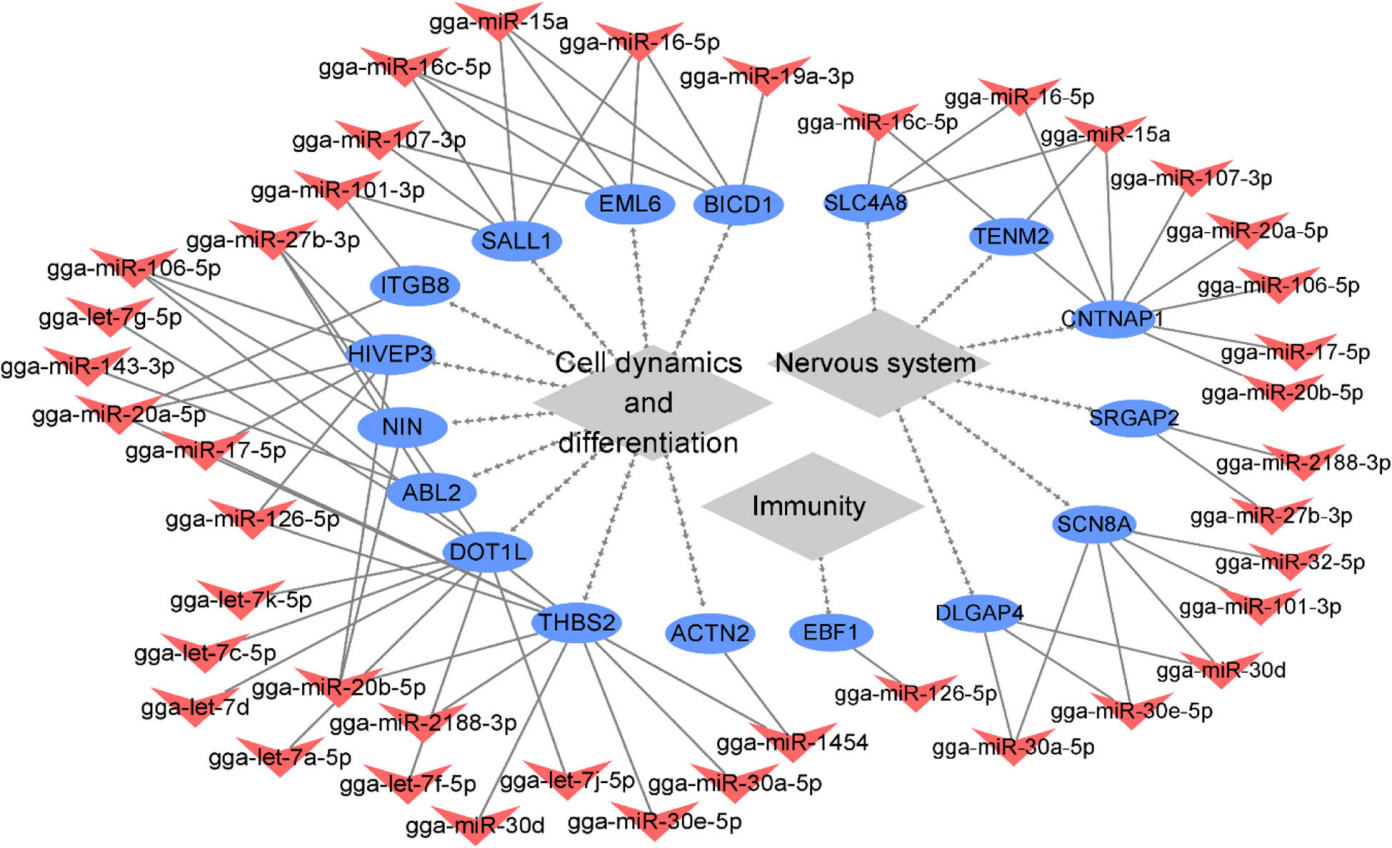
Candidate biomarkers of molt. The shapes Ellipse and V represents mRNAs and miRNAs in blood, respectively. Up-regulated genes are represented in red and down-regulated genes are represented in blue

In the last part of the study, results on messenger and micro-RNA were integrated to propose putative biomarkers of molt that can be detected in blood. Integration resulted in the identification of 17 genes presenting DGE or DTU in at least 3 tissues and included in the target genes of the differentially expressed miRNA, and are displayed in Fig. 8 (Additional file 10: Table S10). All of these genes were target of up-regulated miRNAs in blood, and accordingly, down regulated in blood mRNA. One gene only, *CNTNAP1*, was found in DGE or DTU in 4 different tissues, all other genes being present in 3 tissues. If eight of the genes (*SCN8A*, *SLC4A8*, *HIVEP3*, *EBF1*, *TENM2*, *DOT1L*, *SRGAP2* and *NIN*) were found to be down-regulated in all tissues, expression pattern did not always match between blood and tissues. The genes *DGLAP4* and *ABL2* were down –regulated in blood and presented DTU in the other tissues, while *ATCN2* was up-regulated in both meninges and bone marrow. Finally, *CNTNAP1*, *EML6*, *ITGB8*, *THBS2*, *BICD1* and *SALL1* presented a mixture of up-and down expression or DTU in the various tissues. The function of these genes was inferred by browsing the literature and GeneCards. Interestingly, all of these genes were found to be implicated either in cell dynamics or differentiation (*NIN*, *DOT1L*, *EML6*, *ITGB8*, *THBS2*, *BICD1*, *ACTN2*, *HIVEP3*, *ABL2*, *SALL1*), nervous system (*CNTNAP1*, *SRGAP2*, *DLGAP4*, *TENM2*, *SLC4A8*, *SCN8A*) or immunity (*EBF1*), functions subjected to intense remodeling during molt.

## Discussion

Annual molt is a crucial physiological event in the life cycle of birds that deeply affects numerous tissues, but whose effects on the organism remain insufficiently elucidated, especially at the level of gene expression. Strengthened by an extensive sampling of various chicken tissues and by the availability of blood mRNA and miRNA sequencing in both males and females, the present study provided a powerful framework to extensively investigate the impact of natural molt on Ginkkoridak transcriptome. Through this study, we provide valuable elements of discussion to the following questions: What is the global impact of molt? Is the effect of molt on blood gene expression different according to sex? Does the ‘signature’ of molt differ across tissues belonging to different histological entities or different physiological function? On the contrary, are there genes whose expression is invariably altered in response to molt across all tissues? The following paragraphs will sequentially try to answer these questions.

Molt was globally associated to a down-regulation of gene expression in most tissues, excepted for female blood, meninges, shin and tail skins and billiducts, in which an up-regulation was mostly observed. The extent of gene expression changes induced by molt was also highly variable from one tissue to another, with as much as 5202 DGE and DTU genes in male blood and as few as 6 DGE and DTU genes in the seminiferous ducts. No other study ever reported the effect of molt on gene expression in any of the tissues used in the present study so that comparison to similar data is for the time being impossible. However, molt-induced gene expression changes were quantified through microarray in laying hens’ reproductive tract [4], that evidenced mostly down regulation during the first week of molt, followed by up-regulation in the later stages (Day 30–35). By contrast to this study, our works didn’t evidence extensive molt-induced gene expression remodeling in the male chicken reproductive tract. Gene expression thus appears to differ based on the tissue concerned and on the stage of molt.

The second part of the present study consisted in determining whether the effect of molt on blood gene expression was different according to sex. A discrepancy in gene expression was indeed observed, with males being substantially more impacted than females in terms of number of differentially expressed genes and differentially expressed miRNAs. What is more, molt was mostly associated with gene over-expression in females while gene expression was largely decreased in males. To further decipher these results gene ontology was performed on the genes being down-regulated in both sexes but no pattern emerged. However, genes down-regulated in males and up-regulated in females appeared to be related to terms linked to chromosome structure. A legitimate questioning arising from these results would be whether these differences could correspond, at least partly, to sex-biased expression. Males are subject of stronger sexual selection than females [24] and molt corresponds to the renewing of appendages, which heavily conditions sexual dimorphism in birds. One hypothesis worth consideration is that the higher impact of molt on male blood gene expression could result from this stronger sexual selection. Though this hypothesis is tempting, sex biased gene expression is known to be highly different according to the tissue considered [25] and the data at our disposal (both sexes RNA-Seq data was available in blood only) didn’t enable further investigation.

The third part of the analysis consisted in determining whether the effects of molt differed across tissues belonging to different histological entities or physiological functions. As extensively described above, this analysis was performed after clustering the tissues based on their gene expression profile. Unsurprisingly, the groups obtained roughly matched the histological profiles of the tissues. Globally, the gene expression modifications occurring during molt found in each tissue clusters were in coherence with the type of the tissues considered: ontology terms related to epidermal differentiation and genes directly related to skin and feather biology were found in the epithelial cluster, genes related to synapse were found in the nervous tissues cluster and terms related to actin and muscle contraction were found in muscle tissues. Of greater interest here, the modifications occurring during molt reported here at the transcriptome level in various tissues confirmed the changes observed at the phenotypic level: immunity, cell dynamics, dermis and nervous system are all putatively impacted by molt [2]. One of the early hypothesis formulated about the neurobiological control of molt was that this life event could be associated to a switch of the autonomic nervous system from the parasympathetic nervous system to the sympathetic nervous system, traducing a state of stress for the organism [2]. Our results are compatible with the hypothesis of an impact of molt on the autonomic nervous system. In the nervous tissues cluster, composed of the cerebrum, cerebellum, hypothalamus, eye and meninges samples, genes in ontology terms related to cholinesterase and acetylcholinesterase activities were found altered by molt. Acetylcholine is a neurotransmitter that has been reported to act as a neuromodulator and to alter neuronal excitability [17], but, most importantly, acetylcholine, along with norepinephrine and epinephrine mediate all the effects of the autonomic nervous system on the target organs: skeletal muscle, heart and digestive organs among others [26]. Accordingly, tissues from the nervous cluster were not the only organs in which terms related to the nervous system were enriched: lung and bone marrow gene expression alterations also exhibited patterns related to nervous system. Aside from the aforementioned elements, few genes related to the neuroendocrine control of molt were found altered in the various tissues considered in the present study. The ‘Photoreceptor inner segment’ and ‘Exocrine system development’ GO:CC and GO:BP terms were found enriched in the meninges; the *PER2* and *CRY2* genes, related to the control of circadian clock in chicken [27, 28] and the *CRH* and *PTHLH* genes, coding corticotropin-releasing hormone for parathyroid hormone-related protein, respectively, were found increased in the shin skin during molt and might be related to the neuroendocrine control of molt [8]. Our results also support the hypothesis of an intense remodeling of muscle tissue and changes in the energy metabolism during molt. Muscle tissues presented an altered gene expression profile relatively distinct from other tissues, bone marrow excepted. Briefly, muscle tissues and especially heart, that was substantially impacted by molt, presented some evidences of muscle cell motility modification and ‘cell crawling’, putatively pointing to a renewing of muscle tissue [21, 22]. Immunity-related gene expression alterations were particularly important in the lungs compared to other tissues. Lungs being a prevailing entry route of pathogens in chicken, it is somehow logical that immunity-related genes could be modified intensely in this organ during molt [29]. Besides lungs, immunity in the intestinal tract has also been reported to be altered during molt at the phenotypic level [30]. Unfortunately, we couldn’t assess this fact on the transcriptomic level due to the absence of sampling on intestinal tissues. Finally, at the ‘tip of the iceberg’, skin and plumage were intensely affected by molt. Appendages-related tissues were affected by molt quite differently from the other groups. In particular, 56 genes related to keratins were found to for the most part overexpressed in shin skin, earlobe and tail skin. Most of these keratin-related genes could be mapped to the β-keratin group, proteins absent in mammalians but that constitute a key component of feather, scale, claw and beak of birds, and whose diversification is at the origin of the unique properties of feathers [31]. Up-regulation of β-keratins genes is not surprising as the ‘during molt’ samples were collected at the stage when new feathers were the smallest, stage at which the development of feathers is expected to be intense. Most of the β-keratins genes are encoded in a gene cluster named Epidermal Differentiation Complex (EDC), which gathers many of the structural components of the cornifying keratinocytes [32]. The EDC encoded proteins, in conjunction with other non-EDC-encoded proteins define a spatio-temporal cell differentiation program, which determine the body of the feather during the morphogenesis and maturation of the feathers [33]. Some of the most up-regulated genes in tail skin, *EDCH3* and epidermal differentiation protein starting with MTF motif 3-like, along with *EDBETA,* one of the 3 genes most constantly up-regulated across skin tissues (earlobe, shin and tail skins), were part of the EDC. Collecting samples from the feather follicles would have bridged the gap and possibly allowed us to integrate more evidences on the mechanisms of feather renewing, starting from the hormonal control in the cerebral regions, through blood and to the skin tissues. If the unavailability of the feather follicle tissues is regrettable for integration of the data, this topic has already been more extensively described in the literature than the impact of molt across other somatic tissues, and reviewed by Chen et al. [34].

Finally, the core of the paper consisted in identifying genes whose expression would be invariably altered in response to molt across the widest range possible of tissues. This was performed using a two-step strategy: first the genes showing DGE and DTU having the highest ‘overlap score’ across tissues were investigated as potential markers. In the second step, profit was taken from the concomitant availability of mRNA and miRNA data to further refine the analysis. Target prediction of miRNA is indeed known to generate large amount of false positive. Integration of mRNA and miRNA increases reliability of results as it selects transcriptional (mRNA) and post-transcriptional (miRNA) regulations that converge in the same direction (up- or down-regulation) for the concerned genes. Micro-RNA-messenger RNA integration analysis is thus advisable for discerning the precise regulatory systems. Two major patterns emerged from the genes showing DGE or DTU in the greatest number of tissues: two genes, *U1* and *U4*, coding for the spliceosome and two other genes, *RPL21* and *RPL23*, coding for ribosomal proteins. The spliceosome is a complex composed of five small nuclear (sn) RNA, namely U1, U2, U4, U5 and U6, to which around 100 proteins are associated. This complex is responsible for alternative splicing, the process which enhances the diversity of proteins produced by the genes [13]. Interestingly, the *U1* and *U4* genes were down-regulated in all the tissues in which their expression was altered, except for blood in which they were up-regulated. This change in the regulation of splicing during molt ask for legitimate interrogation on whether this fact could have had any influence on the differential transcript usage observed in some of the tissues studied and would necessitate further consideration. Ribosomal proteins, aside from playing a role in ribosome biogenesis and protein translation, have also been proved to regulated diverse cellular processes, such as apoptosis, cell proliferation or cell migration [35]. Though inferring any conclusion in the present case is highly speculative, these functions might converge with the intense remodeling observed across tissues during molt. The second and final step of this study consisted in integrating mRNA and miRNA data to identify reliable biomarker genes of molt. A total of 17 genes resulted from this approach. Of great interest, all of the candidate marker genes could be related to processes associated with known consequences of molt: cell dynamics, nervous system or immunity. With regard to cell dynamics, the *EML6* gene has been reported to mediate regulation of stability of microtubules [36] while *BICD1* is implicated in dinein motor activity [37]. The genes *ITGB8* and *THBS2* are involved in vascularization [38, 39], a process that has been shown to be altered during molt [2]. Furthermore, *HIVEP3* that functions as transcription factor is related to modulation of immunoglobulin gene rearrangement, cell survival and TNF-signaling as well as osteoblastic bone and tumor formation [40]. Interestingly, molt was reported to be associated with osteoporosis in birds [2], a phenomenon in accordance with the down-regulation in the *HIVEP* gene found in blood, heart and breast muscle tissues in the present study. In total, six of the candidate biomarkers genes were found to have a role within the nervous system. The genes *TENM2, SLC4A8* and *SCN8A* respectively encode a synaptic cell adhesion molecule [41], a membrane protein regulating pre-synaptic pH [42] and a mediator of neuronal excitability [43]. Additionally, *CNTNAP1,* plays a central role in the formation of paranodal junctions in myelinated axons [16], *SRGAP2* negatively mediates neuronal migration [44] and *DLGAP4* modulates neuronal cell signaling by organizing synapses [45]. Lastly, the *EBF1* gene, found to be downregulated in blood, meninges and heart, is a transcription factor that has been shown to be essential in the commitment of the B cell lineage [46]. The present study thus corroborates at the gene expression level in natural molt the works of Holt (1992) [6], who reported a transient decrease in the number of the B cell during induced molt. Molt might thus be associated to altered immune response in case of a new pathogenic challenge. If the candidate biomarker genes proposed above provide valuable information, further inferring of their role in molt based on our data solely would be hazardous because of 1) the pattern inconsistency found across tissues 2) the wide range of effects some of these genes encode. The *DOT1L* gene, for example, has been shown, in mouse, to mediate effects as diverse as proliferation of embryonic stem cells, chondrogenesis, cardiac development and reprogramming of somatic cells [47]. Another layer of complexity is that the effect of these pleiotropic genes is likely to vary across tissues. Further validation of the effect of the individual aforementioned genes on the molt phenotype is thus advisable.

## Conclusion

To conclude, the present study took advantage of an extensive gene expression dataset to decipher the impact of molt a physiological event of great importance in the life of birds that remained poorly investigated. The integration of messenger and micro RNAs further increased the reliability of the potential role of miRNA and candidate biomarkers of molt proposed here. This study overall confirmed at the transcriptome level the effects of molt in rooster reported at the phenotype level, particularly with regards to changes occurring on the autonomic nervous system. We conclude that the results of this study can be utilized as a valuable resource in future transcriptome analyses of chicken and provide novel insights into molt, one of the crucial processes in avian lifecycle.

## Methods

### Messenger RNA

#### Experimental design and sampling process

A total of 20 Ginkkoridak, 10 males and 10 females, were used in this study. Animals were randomly selected in their conservancy farm (Pungdong) located in the surroundings of Seoul, South Korea. Blood (males and females) and tissue samples (males only) were collected, before the onset of annual molt (in July, 5 males and 5 females, 24 months of age) and during molt (in October, 5 males and females, 30 months of age). During molt sampling was performed when the renewed feathers are the shortest. Sample tissues were not available for one of the before-molt males, which had been used for the establishment of Ginkkoridak reference genome. A total of 24 tissues were sampled for the study, which are displayed in Fig. 1A. Blood samples were collected from the brachial vein using minicollect® K3EK3EDTA tubes for total RNA extraction. Tissue samples were collected after euthanasia of the animals and RNA was extracted within 24 h. Animal specimens were euthanized in CO2 chamber with accordance to regulations of EU law and the American Veterinary Medical Association (AVMA). Ginkkoridak samples were collected under a strict supervision of a veterinarian following good animal practice. All experiments and procedures involving animals were approved by the Institutional Animal Care and Use Committee of the National Institute of Animal Science (NIAS) of Republic of Korea under approval number NIAS2018268.

#### RNA isolation and quality assessment

RNA was extracted from whole blood (3 mL collected per animal) in both sexes. Tissues samples were collected in male only using TruSeq seq standed Total RNA sample prep kit (300 ng collected per sample).

#### Library preparation and sequencing

Each sequenced sample was prepared and sequenced by Macrogen (Seoul, Korea). Samples were prepared according to the Illumina (San Diego, CA, USA) protocols: TruSeq Stranded Total RNA LT Sample Prep Kit (Globin) for blood and TruSeq Stranded Total RNA LT Sample Prep Kit (Human Mouse Rat) for tissue samples.

Sequencing libraries were generated according to the TruSeq Stranded Total RNA Sample Prep Guide, Part #15031048 Rev. E (Illumina). Paired-end sequencing was performed using the Illumina HiSeq2500 platform (Illumina), with a read length of 100 bp.

#### Raw reads pre-processing

The raw blood and tissues RNA sequence data was then further processed according to the protocol described below. First, raw reads issued from sequencing were quality checked using the FastQC software [48] and ribosomal RNA contamination was removed using bbduk [49] with the files based on the SILVA database made available by B. Bushnell at https://drive.google.com/file/d/0B3llHR93L14wS2NqRXpXakhFaEk/view?usp=sharing as reference for the ribosomal sequences. After a second FastQC quality check, Trimmomatic [50] was used with TRAILING:20, SLIDINGWINDOW:4:15 and MINLEN:75 options to remove low quality bases and artifact sequences. Quantification of the expression of the transcripts was performed using Salmon [51]. Salmon is a pseudo-aligner providing a fast and accurate estimation of expression while correcting for some of the biases commonly observed in RNA-seq data. The Galgal6 decoy-aware transcriptome first was build beforehand by 1) listing the decoys from the chicken genome sequence data (assembly GRCg6a) downloaded from Ensemble 2) building the transcriptome by merging the cDNA and ncRNA sequences downloaded from the chicken genome assembly GRCg6a on Ensembl and 3) concatenating the genome to the end of the transcriptome. Then, the decoy-aware transcriptome was indexed using the Salmon command index, with providing the decoys list as input (option –d). Finally, the samples’ transcripts were quantified by running the Salmon quant command, using the following options: –gcBias to correct for GC content bias in the samples and –validateMappings to perform selective alignment. Due to a substantial part of reads mapping to introns, the overall alignment rate to the transcriptome varied between 37.4 and 54.0% across samples for blood and between 28.0 and 92.0% for tissue samples. Finally processed read numbers varied between 10.3 and 28.8 million for blood and 4.3 and 59.6 million for tissues.

#### Differential gene expression

Experimental groups differed for the type of sample considered. As mentioned above, blood samples were available on a total of 20 different individuals, 10 males and 10 females, 5 males and females sample being collected before molt and the remaining 5 males and females being collected during molt. These blood samples could thus be used to model a total of 4 comparisons: 1) during and before molt in males 2) during and before molt in females 3) males vs females before molt and 4) males vs females during molt. The various tissue samples (*n* = 24) were available on 9 males only, so that only during and before molt gene expression and transcript usage could be compared. The before and during molt tissue groups comprised 4 and 5 biological replicates, respectively.

Transcripts counts from Salmon were imported into R using the tximport package [52]. Genes with no counts or a single count across all samples were removed to improve computation time. Differential gene expression analysis for each experimental group was performed using the R package DESeq2 [53], which provides adjusted *p*-values, controlling the false discovery rate (FDR) using Benjamini and Hochberg’s method. Genes with an adjusted p-value < 0.05 and |log2FoldChange| ≥ 1 were considered as differentially expressed.

#### Differential transcript usage

As a complement, the transcript-level estimated counts imported using tximport were used to estimate differential transcript usage in a two-step procedure. The R package DEXSeq [15, 54] was first used to compute the gene adjusted p-values and the transcripts p-values. In a second step, the R package stageR [55] was implemented to control the overall false discovery rate (OFDR). An OFDR threshold of 5% was applied, meaning that the set of genes considered as exhibiting DTU are expected to contain “no more than 5% of either genes which have in fact no DTU or genes which contain a transcript with an adjusted p-value less than 5% which do not participate in DTU” [56].

#### Tissues clustering

In order to provide the most comprehensive functional enrichment analysis of the data given the elevated number of different tissues involved in the study, tissues were grouped based on a prior clustering. First, counts of genes presenting DGE or DTU in at least one of the tissues were normalized using the varianceStabilizationTransformation function from the DESeq2 package in order to get approximately homoscedastic data and to normalize for library size. The R package FactoMineR [57] was the used for clustering. The first step consisting in denoising the data through Principal Component Analysis (PCA), which reduces the dimension of the data into few continuous variables encompassing most of the information. The HCPC method from the same package was then employed. Briefly, hierarchical clustering was performed using the Ward’s criterion on the PCA components; the number of clusters was chosen by cutting the hierarchical tree according to inertia gain and the initial partition was refined through K-means clustering. The clustering dendrogram was modified with the R package circlize [58].

### Micro RNA

#### Small RNA isolation and quality assessment

Small RNA was extracted from whole blood (3 mL collected per animal) in both sexes using TruSeq Small RNA library prep kit.

#### Library preparation and sequencing

All small RNA samples were processed by Macrogen (Seoul, Korea). Samples were prepared according to the Illumina protocols (San Diego, CA, USA, TruSeq Small RNA Library Prep). Sequencing libraries were generated according to the TruSeq Small RNA Library Prep Guide, Part #15004197 Rev. G (Illumina). Single-end sequencing was performed using the Illumina HiSeq2500 platform (Illumina), with a read length of 50 bp.

#### Raw reads pre-processing

The quality of the raw reads was first checked using FastQC [48], followed by a removal of rRNA using bbduk [49]. After a second quality check (FastQC), the Illumina Small RNA adapters were trimmed from the sequences using Trimmomatic [50] with the following options: LEADING:3, TRAILING:3, SLIDINGWINDOW:4:15 and MINLEN:18. Then Cutadapt [59] was used applying option M:32, so that only the reads with a length comprised between 18 and 32 nucleotides were kept. After a final FastQC quality, check alignment was performed using Bowtie [60] and mirDeep2 scripts [61]. More precisely, the Bowtie command “bowtie-build” was first used to index the chicken genome assembly GRCg6a downloaded from Ensembl. The mirDeep2 mapper.pl script was then used to process reads and map them to the reference genome provided. The miRNAs were then identified and quantified using the mirDeep2.pl wrapper function, which relies on miRBase v22.1 for identification of miRNA sequences. As Bowtie allows multi loci mapping of reads, which is likely to generate bias, an expression calibration step was performed by dividing reads that multi-map to mature miRNA by the ratio of whole reads count that are mapped on the miRNA’s precursor. In addition, only reads perfectly matching the reference sequence were kept. The expression data resulting from the mapping step was expressed as RPM.

#### Prediction of target genes and identification of miRNA-mRNA pairs

The DGE analysis was implemented on the miRNA aligned reads, with the same methodology as described for mRNA blood samples (DESeq2, model with 4 comparisons and FDR adjusted *p*-value). The resulting up- and down-regulated miRNA lists were used as input in miRDB [62] to predict miRNA target genes. Only miRNA having less than 1500 putative target genes and only target genes associated with a threshold score > 80 were consider for further analyses. Spearman’s rank correlation analysis was conducted to identify negatively correlated miRNA-mRNA pairs. The target genes having a significant and negative correlation values (adjusted *P*-value (FDR) < 0.05) and that were part of the differentially expressed mRNAs were considered for further analysis. In addition, the network of significant miRNA-mRNA pairs was constructed and visualized using Cytoscape [63].

### Integration of the results and functional enrichment analysis

In order to further refine the effects of molt, we integrated the results and selected the strongest candidate genes present in the significant miRNA-mRNA pairs obtained on blood in male chicken and in DGE or DTU in at least three different tissues (blood included). Because molt could also have been associated to changes specific to individual tissues or histological group of tissues, functional enrichment analysis was performed on various combinations of tissues. Functional enrichment analysis was performed using g:Profiler [64] with FDR < 0.05 or 0.01 depending on the input gene set. Large gene ontology results were further filtered using REVIGO [65] with allowed semantic similarities (SimRel) < 0.7 (genes ontology terms < 100) or 0.5 (more than 100 GO terms).

## Supplementary Information

**Additional file 1: Table S1.** Top 5 DGE (under and over-expressed) and DTU genes across the 23 Ginkkoridak tissues.

**Additional file 2: Table S2.** DGE (under and over-expressed) and DTU genes present in at least 3 tissues in male Ginkkoridak.

**Additional file 3: Table S3 A.** Expression pattern for genes altered during molt in male and female Ginkkoridak blood. **Table S3 B.** Expression pattern for genes up-regulated during molt in both male and female blood.

**Additional file 4: Table S4 A.** Expression pattern for genes altered during molt in male Ginkkoridak in at least two of the epithelial tissues cluster (craw, tongue, earlobe, shin skin, muscle and tail skins). **Table S4 B.** Expression pattern for genes altered during molt in male Ginkkoridak in at least two of the nervous tissues cluster (cerebrum, cerebellum, hypothalamus, eye, meninges).

**Additional file 5: Table S5 A.** Overall mapping data of miRNA in Ginkkoridak blood. **Table S5 B.** Sequence length distribution.

**Additional file 6: Table S6 A.** Identified miRNAs from all samples in Ginkkoridak blood. **Table S6 B.** Differentially expressed miRNAs in Ginkkoridak. (Before molt VS During molt).

**Additional file 7: Table S7.** Target genes of the Differentially Expressed miRNAs.

**Additional file 8: Table S8 A.** Overlap genes between miRNA Target genes & mRNAs (oppisite direction of expression between miRNAs and mRNAs). **Table S8 B.** Significant miRNA-mRNA pairs (Spearman’s Rank Correlation Analysis).

**Additional file 9: Table S9.** Expression pattern for significant overlap genes altered during molt in male and female Ginkkoridak blood.

**Additional file 10: Table S10.** Summary of candidate biomarkers of molt.

## Data Availability

The datasets (blood) generated and/or analyzed during the current study are available in the NCBI repository, http://www.ncbi.nlm.nih.gov/bioproject/707212. The other datasets used and/or analyzed during the current study available from the corresponding author on reasonable request.

## Abbreviations

DGE: Differential gene expression
DTU: Differential transcript usage
DEM: Differentially expressed miRNAs
